# China's low fertility may not hinder future prosperity

**DOI:** 10.1073/pnas.2108900118

**Published:** 2021-09-27

**Authors:** Guillaume Marois, Stuart Gietel-Basten, Wolfgang Lutz

**Affiliations:** ^a^Asian Demographic Research Institute, Shanghai University, Shanghai, China;; ^b^Wittgenstein Centre for Demography and Global Human Capital, University of Vienna–VID/ÖAW–International Institute for Applied Systems Analysis, 2361 Laxenburg, Austria;; ^c^Division of Social Science, The Hong Kong University of Science and Technology, Hong Kong SAR, China

**Keywords:** fertility, China, aging, human capital, dependency ratio

## Abstract

China’s low fertility is often presented as a major factor which will hinder its prosperity in the medium to long term. This is based on the assumed negative consequences of an increasing old-age dependency ratio: a simplistic measure of relative changing age structures. Based on this view, policies to increase fertility are being proposed after decades of birth restriction policies. Here, we argue that a purely age structure–based reasoning which disregards labor force participation and education attainment may be highly misleading. While fertility has indeed fallen to low levels, human capital accumulation has been very strong—especially among younger cohorts. Factoring in the effects of labor force participation and educational attainment on productivity, a measure called “productivity-weighted labor force dependency ratio” can more accurately capture the economic implications of demographic change. When using this ratio, a much more optimistic picture of the economic (and social) future of China can be envisaged.

## Significance

China has the world’s largest national population and is rapidly catching up with the United States in terms of having the status as the world's largest economy. In this context, recent reports about unexpectedly low levels of fertility have given rise to speculation that the resulting population stagnation/decline and rapid aging may pose a major obstacle to continued prosperity in the future. We show that, depending on the indicator of demographic dependency used, the future may look very different. When associated with rapid increases in human capital, low fertility rates may not pose such a significant obstacle to continued development over the coming decades. Whether this will be the case may have profound geopolitical and global economic consequences.

Low fertility appears to be very much back on the scientific and policy agenda. Recent years have seen total fertility rates (TFRs) in some industrialized countries reach historic lows, even in unexpected settings such as Finland and the United States (1). Most recent data on births and pregnancies indicate further declines in the context of the current pandemic, with China reporting a steep decline in births in 2020, causing great alarm in state media (2). The standard narrative links such low fertility to increases in the pace and scale of population aging with assumed negative consequences for the economy and the sustainability of public services as well as more fundamental issues such as gerontocracy and intergenerational fairness, geopolitical strength, or even the continued existence of a culture.

Around the world, deep concerns about this “demographic time bomb” are increasing, and the European Commission has even appointed a Vice President for “Democracy and Demography.” However, for governments, it is difficult to influence population aging directly (3). Despite being generally debunked, the concept of “replacement migration” (compensating a declining number of younger workers through immigration) lingers as a possible solution. More commonly, though, governments have tried to stimulate the fertility rate through more or less explicit policy interventions. The general failure of these interventions has led to the dual metaphors of the “population time bomb” and the (deeply offensive) “silver tsunami”: slow-burning yet inevitable events which will threaten societies in the long run.

This narrative has been familiar in Europe, Japan, and other parts of East Asia (4). Recently, however, much attention has been paid to China’s demographic travails. Identifying Chinese fertility levels is fraught with difficulties, with ranges being derived from different sources and agencies (3, 5, 6). According to the China Population and Development Center, China’s TFR in 2019 was 1.45 (7), while evidence from the 2020 Census suggests it may have fallen to 1.3 (8)—lower than that of Japan.

In response, the Central Government has introduced a national “three-child policy,” and various local governments have been active in designing interventions to stimulate the fertility rate within the existing boundaries of the policy (3). The recent relaxations of the birth control policies have had relatively little impact, and fertility preference surveys reveal a sub–two-child norm among large segments of the population (3, 9–11), including the “floating population” of internal migrants (12). In this context, there is little expectation that the recent relaxations to permit more than two children per family may make any meaningful change to the overall TFR.

In many analyses, this “gloomy demographic forecast” for China is translated into economic (and even geopolitical) disaster. It is suggested that China is “getting old before it gets rich” and may, thus, not be able to catch up economically with the United States as previously assumed. Some headlines even present such circumstances as an existential threat. One 2021 newspaper headline, for example, declared that “Population decline could end China’s civilization as we know it” (13).

These prognostications are partly based on a simplistic and misleading concept of age dependency that assumes that everybody above age 65 is an economic burden and every adult below this age is an (equally) productive asset. The so-called “age dependency ratio” (ADR) in contemporary China is around 38, meaning there are 38 “dependents” aged over 65 or fewer than 15 per 100 “workers” aged between 15 and 64 (14). By 2050, this is forecast to rise to 67, as high as many contemporary European settings (1). Demographers have long argued, however, that this measure is misleading for addressing the economic implications of aging because it ignores dynamic changes in health and longevity as well as in labor force participation, education, and productivity (15). When alternative measures of aging are applied to China, the overall picture of a “crisis” as presented by traditional measures looks much more manageable (16–18).

## Considering Further Dimensions of Demographic Change

As the science that studies changing population size and structures, demography is not only concerned with age structures but also with other societal characteristics such as changing structures of educational attainment (19) and labor force participation by age and sex (20). Here, we show that the future of China looks very different if such a multidimensional demographic approach is taken that also reflects the massive improvement of human capital that China has experienced, and will continue to experience, because the younger generation today is already much better educated than the older generation and will replace it through the predictable mechanism of “demographic metabolism” (21).

A recent study has taken such a multidimensional approach to aging by stratifying the population of the (then) 28 European Union (EU) member states by not only age and gender but also immigration status, labor force participation, and educational attainment, which is used as a proxy for productivity, reflecting the fact that not all individuals are equally productive (20). The study then proposes alternative measures to the ADR, such as the “labor force dependency ratio” (LFDR) (which compares those economically active and inactive of all ages) and a “productivity-weighted labor force dependency ratio” (PWLFDR) (which approximates differences in productivity through wage differentials associated with various levels of educational attainment). The study shows that these alternative dependency measures increase much less than the ADR, and when combined with Canadian-style immigration policies, they even lead to a decline in economic “dependency” in the EU over the coming decades.

When applying these alternative measures of LFDR and PWLFDR to the future of China, the situation is complicated by significant uncertainty about the baseline fertility measures (3, 5). As to the future trends of the TFR, one could think of two opposing scenarios: 1) assuming that China will follow a similar path as South Korea, where the TFR falls to around 0.8, and 2) that it will follow the United Nations assumptions of a recovery to around 1.7. Rather than taking a position on what the TFR in China will be, we simply calculate the implications of two alternative cases—a “high” scenario of 1.7 and a “low” scenario of 0.8.

Projections are performed with a dynamic discrete-time microsimulation model simulating life events of individuals (fertility, mortality, migration, education transitions, and participation in the labor force) with Monte Carlo experiments. The base population in 2015 is synthetically built based on the Wittgenstein Centre for Demography and Global Human Capital (hereafter "Wittgenstein Centre") population estimates by age, sex, and educational attainment and contains about 700,000 cases (14). Assumptions in regard to mortality (by age, sex, and education), migration (by age and sex), and expansion of education (by cohort and sex) are those from the medium scenario (SSP2) of the Wittgenstein Centre projections (14), themselves built from statistical modeling of past trends and expert judgments (*SI Appendix*, Table S1 for a summary of assumptions). The model is run with two fertility assumptions, assuming a constant TFR of either 0.8 or 1.7 children per woman starting from 2015. The labor force participation is modeled from a cross-sectional survey using parameters from a logit regression (Eqs. **1**–**3**) estimated with microdata of the Chinese General Social Surveys (CGSS) from 2010 to 2017 (see *SI Appendix*, Table S2 for model details). Those parameters are assumed to stay constant throughout the projection. However, as the statistical model explicitly accounts for the higher propensity of working for highly educated women (*SI Appendix*, Fig. S1), the expansion in education dynamically increases the general labor force participation among workers. The productivity weights by education level are estimated from Poisson regression parameters on the salary variable of the CGSS 2010 to 2017, excluding the population out of the labor force and controlling for age, sex, and year (see Eq. **5**). Those weights, ranging from 0.36 for workers with no education to 2.03 for workers with a postsecondary education, are used to factorize the workers in the denominator in the calculation of the PWLFDR (see Eq. **4**).

## Results

### A Picture of Strongly Improving Human Capital.

**Fig. 1.**
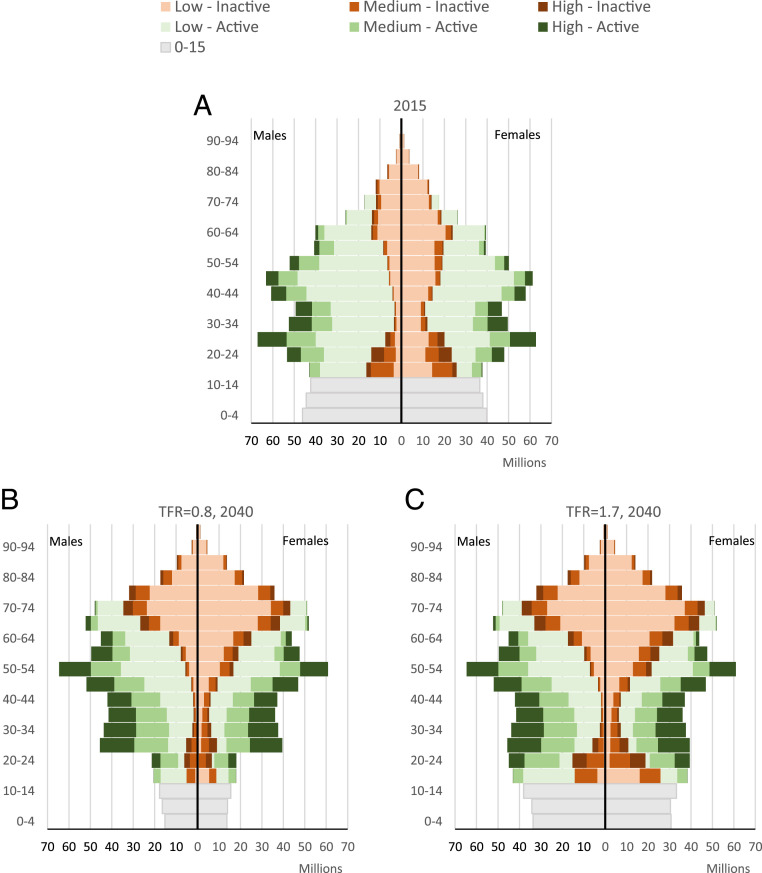
Age pyramid by education (Low = lower than secondary, Medium = upper secondary, and High = postsecondary) and labor force status (Inactive and Active), China, 2015 (*A*); 2040 under TFR = 0.8 scenario (*B*); 2040 under TFR = 1.7 scenario (*C*).

**Fig. 2.**
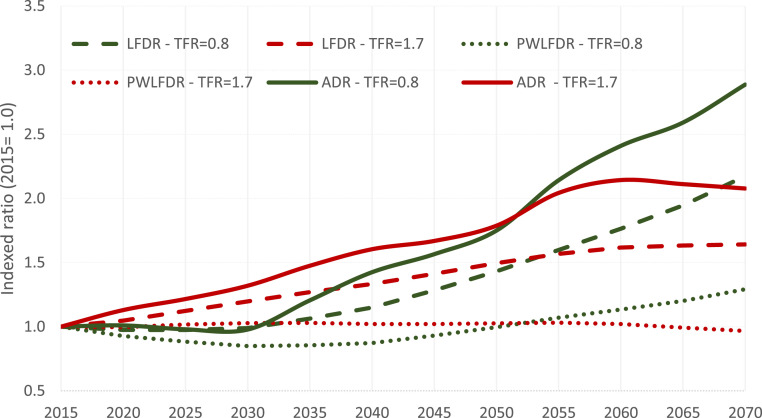
Projected dependency ratios according to two TFRs, China, 2015 to 2070 (2015 = 1). ADR: (population <15 and population 65+)/population 15–64. LFDR: Inactive/Active. PWLFDR: Inactive/Active weighted by productivity (*SI Appendix*).

Fig. 1 depicts the multidimensional demographic changes by age, sex, education, and labor force participation that we will likely see in the human capital of China over the upcoming decades. First, the figure illustrates that by 2040, both fertility scenarios still yield the exact same structures for the adult population above age 25 because the older cohorts have already been born. However, due to the smaller number of children, the lower fertility scenario results in a lower ADR over the coming decades. As seen in Fig. 2, the ADR of the low fertility scenario exceeds the one of the high fertility scenario only around 2050. Hence, even when assessed on the basis of the conventional ADR, lower fertility might give China an economic boost for the next 20 years rather than being an economic burden.

When looking at the projected trends in the LFDR, the pace and scale of aging are slower and lower than when looking at the ADR (Fig. 2), mainly because the increasing education of future adult cohorts of women is likely to boost their labor force participation. In addition, with this expected expansion of educational attainment of the future adult population, the human capital of the working population will improve drastically. Fig. 1 shows that, for both scenarios, the share of the highly educated population among workers, in dark green, is expected to be much larger in 2040 compared with 2015 simply because of the fully predictable fact that the younger, already better educated cohorts will move further up the age pyramid. When transposing this dynamic into the PWLFDR (Fig. 2), there is virtually no increase in forecast dependency up to 2070, even under the low fertility scenario. While the total number of workers will start declining before 2025, those with a high level of education (postsecondary) will keep growing, as new cohorts entering the labor market are much more educated than older ones retiring (*SI Appendix*, Fig. S2).

## Discussion

### A More Positive Outlook Based on Human Capital.

Our simulations show that indicators of dependency which take into account other population characteristics (such as education and productivity) present a very different, more optimistic future for China—even under conditions of very low fertility. This is not to say that the ongoing demographic change within China will be without difficulties. China will almost certainly have challenges associated with managing adjustments to population decline and increasing size of the older population, in particular, if fertility should have levels of 0.8 as currently observed in South Korea (22). China does not yet have adequate pension coverage, particularly in rural areas, and to expand social welfare systems will be more challenging. China has already achieved significant education expansion only rivaled by earlier such expansions in much smaller Singapore and South Korea, but it also needs to ensure that these increases are translated (as we assume) into higher labor force participation and higher productivity per worker. This will require ensuring that the current education system is fit for purpose in terms of producing citizens with appropriate tools and skills and that both society and the economy are properly equipped to allow the economic and social potential of all to be fully unleashed. Numerous studies (23), including the groundbreaking work of Rozelle et al. (24), have identified the many challenges ahead to improving the equality of opportunity of access to high-quality education. Increasing overall investment to enhance the quality as well as coverage of education across the entire country (in both rural areas and for those families in urban and rural areas without local household registration [hukou]) is critical to ensure that no children are left behind. Finally, under conditions of very low fertility, the pace of overall population decline will inevitably increase—especially at younger ages. This will inevitably lead to the urgent reorganization of education provision, for example. Without further reform of the labor market to harness the higher levels of human capital, population decline may also impact overall levels of gross domestic product as well as the potential global/geopolitical influence of the country. Such qualitative challenges should, of course, be factored into any interpretation of the models we propose here, which are based on a reimagining of the concept of “dependency.”

We also recognize the technical limitations of our model. While future improvement in education attainment of the adult population is a near certainty due to the already existing much better education of the younger cohorts, the “demographic dividend” (25), in terms of improving economic performance resulting from this, also depends on institutional and other factors that facilitate the translation of higher skills into higher productivity. In terms of data, not knowing the true baseline TFR is a clear challenge, but our simulations based on two widely differing assumptions show that the derived results are robust. Of course, quality of education is arguably as important as quantity. However, this qualitative dimension of education is indirectly captured in the productivity-weighted labor force, which attributes different weights by education to workers based on their productivity (approximated with salary). In a context in which the quality of high school education is very poor, the difference in salary compared to those with no high school would be minimal. This is not the case for China, where both mean years of schooling and skills in literacy-adjusted mean years of schooling increased rapidly from 1970 to 2015 (26).

Despite these limitations, the results illustrate that the still widely used conventional ADR based on a one-dimensional demographic perspective of age alone gives only a very limited, if not misleading, picture compared to the multidimensional perspective, which pays explicit consideration to labor force participation and skills, two dimensions that are more directly linked to economic performance than age alone. The choice of better and more relevant indicators also has far-reaching implications for longer-term economic and geopolitical considerations. Together, these will matter for policy development because bad measures will likely result in bad policies.

## Materials and Methods

### The Microsimulation Model.

The projection model developed for this research uses a microsimulation approach (27). A microsimulation model starts from a baseline population that consists of individual actors whose characteristics represent the composition of a given population across chosen dimensions. These individual actors are exposed to the risk of a set of events relevant to their state and specific to their own characteristics—death, births of a child (which generates a new actor inside the model), moving to a different region in a country, leaving the country, achieving the next level of education, entering or exiting the labor market, and so on. Transitions between the particular states are determined stochastically with a random experiment (the Monte Carlo method). Microsimulation thus allows us to not only include a larger set of dimensions than the standard multistate population projection models but also to easily handle competing risks.

Microsimulation methods are particularly useful when heterogeneity is of significance in the projection modeling or in the projection outcomes (28). A multistate cohort component method can only handle a limited number of dimensions because the number of cells for the transition matrices corresponds to the multiplication of the number of categories of each dimension. In microsimulation, each additional dimension only adds one new column in the dataset. Furthermore, microsimulation can more easily model events for which behaviors can be better understood at the micro level than at the aggregated level. For instance, having had a child in the past few years could be a major predictor for female labor force participation. At the micro level, this predictor can easily be taken into account, as it only requires adding one column recording the time since the last birth occurred and what value is incremented every year without any complex modeling. The variable can then be used in the modeling of other events using, for instance, relative risks or logit regression parameters.

### Model Properties.

The microsimulation model projects the population of China by age (5-y age groups), sex, education, and labor force status from 2015 to 2070. It is built with the Statistical Analysis System (29) and has the following proprieties:

• Time-based: It simulates the life of all individuals from time *t* to *t* + *a* and then repeats from *t* + *a* to *t* + 2 × *a* and so on up to the end of the time span.
• Discrete time: The model only considers the population at specific points in time (by 5-y steps) without considering what could be happening between those points.
• Stochastic: Events are modeled stochastically using random experiments (the Monte Carlo approach), which involves comparing probability with a random linear number from 0 to 1 in order to determine whether or not the event occurs.

The base population is synthetically built from the Wittgenstein Centre estimates of 2015 by age, sex, and level of education (14). The number of individuals generated represents 0.05% of the population size corresponding subgroups, with an oversampling for subgroups with a population size lower than 10,000 in order to minimize the Monte Carlo error for smaller populations. The resulting dataset for the starting population in 2015 has 700,000 cases. Given the size of the starting population, each simulation run would give approximately the same result for aggregated outcomes such as those analyzed in this paper, thus reducing the uncertainty from the Monte Carlo process to an insignificant level. Therefore, multiple runs are not necessary, as the prediction interval would be close to 0. Only one simulation run is enough to produce robust results.

Since the model allows for immigration, some individuals representing immigrants are added throughout the projection, which are generated following the same statistical rules as the base population, according to their characteristics and numbers established a priori in assumptions. For our projections, about 505,000 cases for immigrants are added throughout the projection from 2015 and 2070. When a woman gives birth, a new individual with the same weight is added to the dataset. Thus, the number of new individuals added from the fertility event varies according to fertility assumptions.

Events are ordered as follows: 1) The mortality from *t* to *t* + 5 is first applied with survival ratios by age, sex, and education. 2) Education transition rates by age, sex, and education are then used for educational shifts. From 0 to 14, the education variable is not applied. At the age of 15 to 19, the cohort is broken down according to the education level reached at this age. Transition rates are then applied, and the final educational attainment is reached at the ages of 30 to 34. 3) For those who survive, emigrants are removed using emigration rates by age and sex. 4) Immigrants, for whom numbers and characteristics are set a priori, are then added. 5) Births are generated with fertility rates by age applied to the exposed population. 6) The labor force status is finally established from age-, sex-, and education-specific rates calculated from regression parameters.

### Demographic Assumptions.

Two scenarios are built concerning the TFRs: either 0.8 and 1.7 starting from 2015 and constant until 2070. All other parameters are the same between the two scenarios. However, given that fertility impacts the population size, the number of emigrants, which are calculated from emigration rates by age and sex, also differs among these scenarios. In *SI Appendix*, Table S1, we summarize those assumptions.

For the mortality, the migration, the fertility schedule, and the education progress, assumptions are those from SSP2 of the Wittgenstein Centre multistate projections (14). Those assumptions were established from statistical modeling of past trends and expert judgments, for which detailed explanations can be found in Lutz, Butz, and KC (30) and Lutz et al. (31). This allows for the use of already validated long-term assumptions for those components of the projection instead of having to build them from scratch. The life expectancy is thus assumed to continue its progression while keeping differences by educational attainment. The mean age at birth is also expected to increase, while the immigration is assumed to stay low volume, thus yielding a negative net migration.

### The Labor Force Participation Module.

Labor force participation rates (*P*) are calculated from a logit regression parameters, estimated with data from the CGSS 2010 to 2017 (population aged 15 to 74; *n* = 57,411). The model is described in Eq. **1**:

$$\text{logit}\left(P\right)=\beta_{s,0}+\beta_{s,1}\text{AGEGR}+\beta_{s,2}{\text{AGEGR}}^{2}+\beta_{s,3}\text{EDU}+\beta_{s,4}\text{EDU}*\text{AGEGR}+\beta_{s,5}\text{EDU}*{\text{AGEGR}}^{2}.$$ [1]

The logit of a probability corresponds to the natural logarithm of its odds. Therefore, the logit of the participation rate (*P*) can be calculated from the parameters, such as

$$P=\frac{\text{exp}\left(\beta_{s,0}+\beta_{s,1}\text{AGEGR}+\beta_{s,2}{\text{AGEGR}}^{2}+\beta_{s,3}\text{EDU}+\beta_{s,4}\text{EDU}*\text{AGEGR}+\beta_{s,5}\text{EDU}*{\text{AGEGR}}^{2}\right)}{1+\text{exp}\left(\beta_{s,0}+\beta_{s,1}\text{AGEGR}+\beta_{s,2}{\text{AGEGR}}^{2}+\beta_{s,3}\text{EDU}+\beta_{s,4}\text{EDU}*\text{AGEGR}+\beta_{s,5}\text{EDU}*{\text{AGEGR}}^{2}\right)}.$$ [2]

Each sex has its own set of parameters and its own intercept. The education includes five categories: “No education” (which includes primary incomplete), “Primary,” “Lower Secondary,” Upper Secondary,” and “Postsecondary.” The age group is included with a quadratic function, allowing it to be modeled with a reverse U-shape with lower participation rates for younger adults still in school and older persons. The interaction of age and education allows the model to take into account that the age pattern in labor force participation varies by educational attainment. The max-rescaled R-Square is 0.365 for the males’ model (c-statistic = 0.840) and 0.271 for the females’ one (c-statistic = 0.758). Parameters are presented in *SI Appendix*, Table S2.

In *SI Appendix*, Fig. S1, we show the predicted rates from the model by age and education for both males and females. In general, labor force participation rates for women are 10 to 15% lower than those of men. Women in their 30s with postsecondary education indeed have rates that are close to those of men. The gender gap exists mainly for individuals with lower education as well as for older adults, as the legal retirement age is lower for women than for men. For both males and females, rates are lower for the populations with no education or primary education for most of adulthood, but they become higher past the age of 60, implying that the retirement also comes later for the low-educated population.

In the microsimulation model, those parameters are used to calculate individuals’ probabilities of being in the labor force. The labor force status (in or out of the labor force) is then determined with a Monte Carlo experiment following Eq. **3**:

$$P\{\begin{array}{c} 1\;F_{t+1}<Z∼\left(\left[0,1\right]\right)\\ 0\;F_{t+1}\geq Z∼\left(\left[0,1\right]\right) \end{array},$$ [3]

where Z∼([0,1]) is a random number uniformly distributed between 0 and 1.

The scenarios produced in this paper assume constant parameters for labor force participation and sector of activity. At aggregated levels, this means that any change in that dimension comes from changes in the population composition.

### PWLFDR

The PWLFDR, introduced by Marois et al. (20), divides the number of inactives (the population out the labor force irrespectively of their age) by the number of people in the labor force, weighted by a productivity factor associated to their educational attainment. Eq. **4** depicts this indicator:

$$\text{PWLFDR}=\frac{I}{\sum_{e=1}^{k}W_{e}*A_{e}},$$ [4]

where *I* is the inactive population; *A* is active population with education level *e*, *e* = *e*1 (no education), … , *e*6 (postsecondary); and *W* is the productivity factor associated to the education level *e*.

The calculation of productivity factors relies on the assumption that in a competitive market, at an aggregated level, the average salary is a good proxy of productivity once a statistical control is performed for demographic variables (32, 33). They are estimated from Poisson regression parameters on the salary variable of the CGSS 2010 to 2017, excluding the population out of the labor force and controlling for age, sex, and year, as expressed by Eq. **5**:

$$\ln\left(\text{WAGE}\right)=\beta_{0}+\beta_{1}\text{EDU}+\beta_{2}\text{AGE}\_\text{GR}+\beta_{3}\text{SEX}+\beta_{4}\text{YEAR}.$$ [5]

Productivity factors associated to education levels are estimated with the natural exponent of *β*_1_ and are presented in *SI Appendix*, Table S2. The reference category (*W* = 1) is set for the category upper secondary. Therefore, a ratio of 1 would indicate there is 1 inactive per productivity equivalent to upper secondary worker.

## Supplementary Material

Supplementary File

## Data Availability

Model codes and parameters are available on request. The base population and assumptions are based on the Wittgenstein Centre for Demography and Global Human Capital population estimates (http://dataexplorer.wittgensteincentre.org/wcde-v2/) and the Chinese General Social Survey (http://cgss.ruc.edu.cn/).
