# Supplementary material for: China's low fertility may not hinder future prosperity
Source: Proc Natl Acad Sci U S A. 2021 Sep 27;118(40):e2108900118. doi: 10.1073/pnas.2108900118 (PMC8501780; doi:10.1073/pnas.2108900118)
Supplement: Supplementary File [file pnas.2108900118.sapp.pdf]

PNAS
www.pnas.org

Supplementary Information for

China's low fertility may not hinder future prosperity.

Authors : Guillaume Marois[1,3], Stuart Gietel-Basten[2*], Wolfgang Lutz[3]

1. Asian Demographic Research Institute, Shanghai University, Baoshan, Shanghai, China.
2. Division of Social Science, The Hong Kong University of Science and Technology, Clear Water Bay, Kowloon, Hong Kong SAR, China.
3. Wittgenstein Centre for Demography and Global Human Capital (University of Vienna, IIASA, VID/ÖAW), International Institute for Applied Systems Analysis, 2361 Laxenburg, Austria. * Corresponding author
***Correspondence to:** sgb@ust.hk

This PDF file includes:

SI Table 1-4
SI Figure 1-3

SI Table 1. Summary of assumptions

		2015	2040	2070
**Life expectancy**				
Women	No education	76.1	79.9	85.0
	Primary incomplete	76.8	80.6	85.7
	Primary	77.5	81.3	86.4
	Lower secondary	79.0	82.7	87.7
	Upper secondary	79.9	83.6	88.5
	Postsecondary	80.7	84.4	89.3
Men	No education	71.3	74.8	79.6
	Primary incomplete	72.4	75.9	80.6
	Primary	73.4	76.9	81.6
	Lower secondary	75.6	79.0	83.7
	Upper secondary	76.8	80.3	84.9
	Postsecondary	78.0	81.4	86.0
**Immigration (5-year)**		97,101	121,965	145,975
**Emigration (5-year)**		~0.7%	~0.7%	~0.7%
**Mean age at birth**		27.9	30.9	31.7
**Educational attainment (age 20-39)**				
Women	No education	0.8%	0.0%	0.0%
	Primary incomplete	3.0%	0.2%	0.0%
	Primary	6.3%	0.2%	0.0%
	Lower secondary	47.4%	18.8%	1.3%
	Upper secondary	21.2%	40.0%	29.3%
	Postsecondary	21.4%	40.8%	69.3%
Men	No education	0.5%	0.0%	0.0%
	Primary incomplete	1.9%	0.3%	0.0%
	Primary	4.9%	0.2%	0.0%
	Lower secondary	48.3%	23.1%	3.0%
	Upper secondary	23.0%	41.4%	37.2%
	Postsecondary	21.3%	35.0%	59.8%

SI Table 2. Regression parameters on the labor force participation

		Males			Females		
		Parameter	SE		Parameter	SE	
**Intercept**		0.823	0.614		-1.774	0.614	
**EDU (ref='Primary')**	No education	-5.452	1.197	***	-1.439	1.197	***
	Lower secondary	-1.022	0.705		-2.126	0.705	
	Upper secondary	-8.096	0.676	***	-3.109	0.676	***
	Postsecondary	-12.274	0.752	***	-7.584	0.752	***
**AGEGR**		0.106	0.023	***	0.155	0.023	***
**AGEGR*EDU**	No education	0.165	0.045	**	0.040	0.045	**
	Lower secondary	0.076	0.028	**	0.112	0.028	**
	Upper secondary	0.428	0.029	***	0.197	0.029	***
	Postsecondary	0.658	0.034	***	0.478	0.034	***
**$AGEGR^2$**		-1.75E-03	2.16E-04	***	-2.16E-03	2.16E-04	***
**$AGEGR^2$*EDU**	No education	-1.19E-03	4.14E-04	**	-1.50E-04	4.14E-04	**
	Lower secondary	-1.11E-03	2.69E-04	***	-1.49E-03	2.69E-04	***
	Upper secondary	-5.06E-03	2.96E-04	***	-2.81E-03	2.96E-04	***
	Postsecondary	-7.65E-03	3.72E-04	***	-6.13E-03	3.72E-04	***
**c**		0.840			0.758		
**Max-rescaled R-Square**		0.365			0.271		
**N**		27,844			29,567		

SI Fig. 1. Predicted labor force participation rate by age, sex and education, China

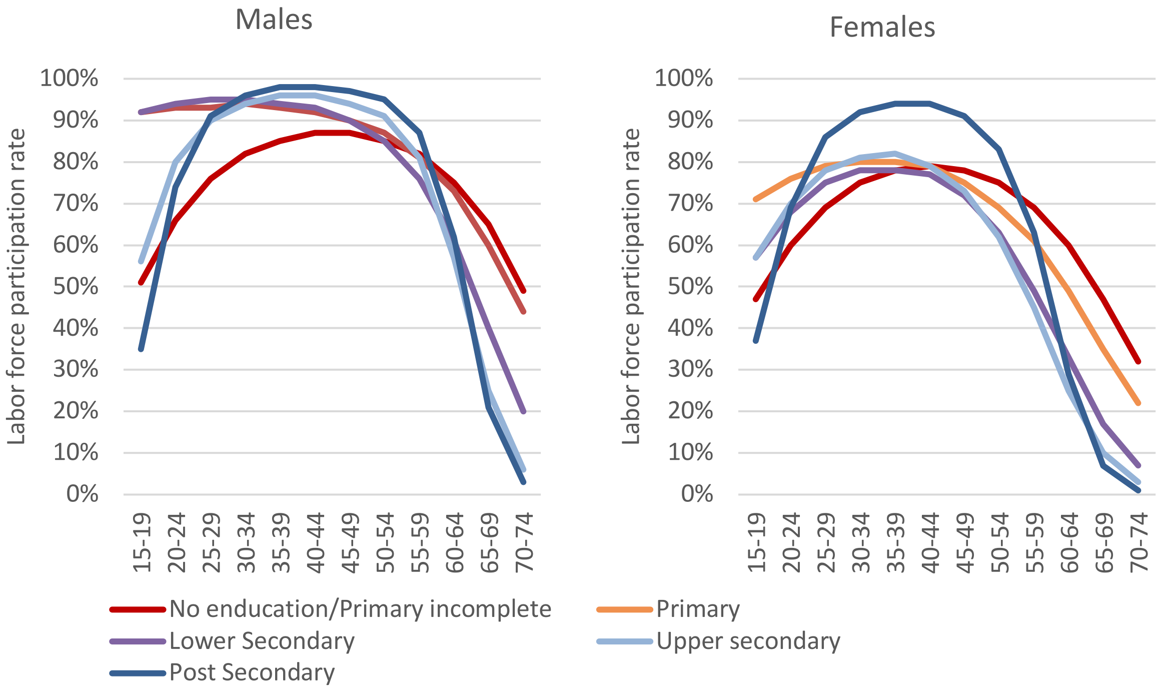

Source: Chinese General Social Survey 2010-2017 (China)

SI Table 3. Productivity factors ($\exp(\beta_1)$) used in the calculation of the PWLFDR

Educational attainment (e)	Factor (W)
No education	0.357
Primary	0.458
Lower secondary	0.694
Upper secondary	1
Postsecondary	2.031

SI Fig. 2. Projected labor force by education, China, 2015-2070

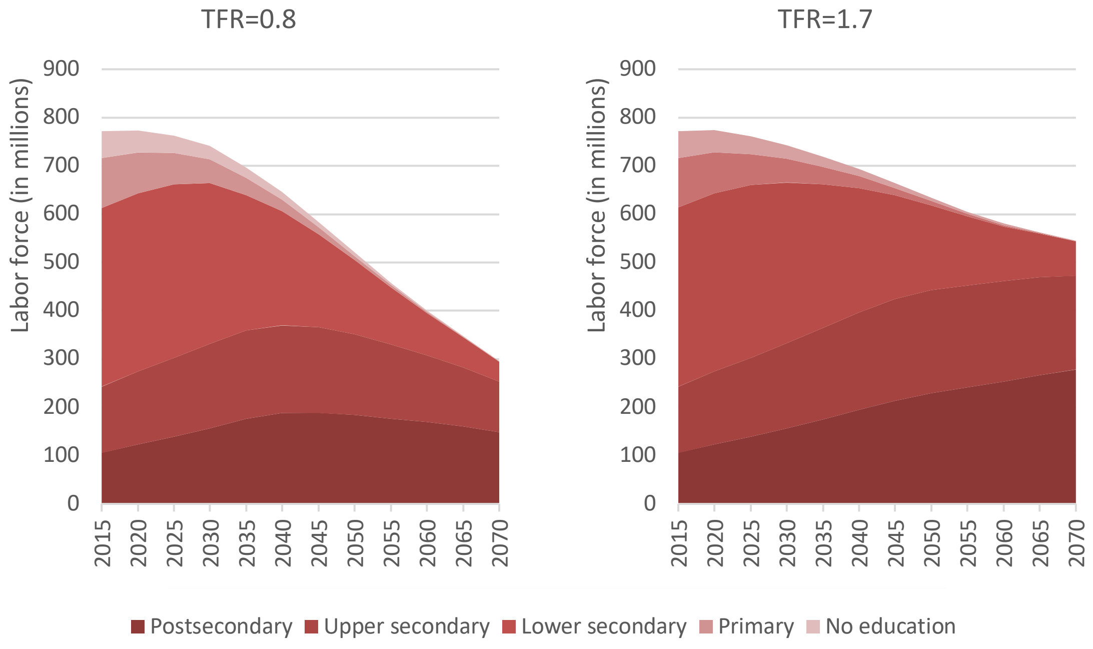

SI Fig. 3. Projected working age size (15-64), labor force (LF) size, and productivity-weighted labor force (PWLF) size, China, 2015-2070

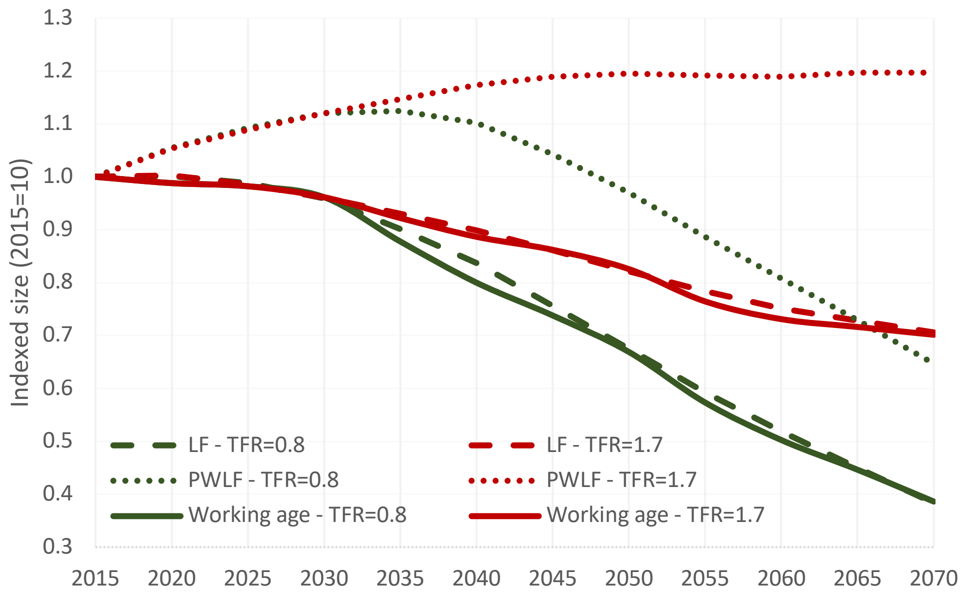

SI Table 4. Projection outcomes, China, 2015-2017

Year	Population (in millions)	Working age population (15-64) (in millions)	Labor force (in millions)	Productivity-weighted labor force (in millions)	Inactive (in millions)	Age dependency ratio	Labor force dependency ratio	Productivity-weighted labor force dependency ratio
**TFR=0.8**								
2015	1397	1015	772	**674**	625	0.377	0.811	0.927
2020	1385	1003	773	**711**	611	0.380	0.791	0.859
2025	1365	997	763	**736**	602	0.369	0.790	0.818
2030	1335	976	741	**755**	594	0.368	0.802	0.787
2035	1296	891	696	**758**	600	0.454	0.862	0.792
2040	1248	812	646	**743**	602	0.537	0.931	0.810
2045	1191	749	584	**703**	607	0.590	1.040	0.863
2050	1126	679	521	**654**	605	0.659	1.160	0.924
2055	1051	582	458	**598**	593	0.807	1.295	0.992
2060	973	510	401	**545**	573	0.908	1.430	1.052
2065	895	453	347	**492**	548	0.976	1.577	1.113
2070	818	392	296	**436**	522	1.088	1.762	1.198
**TFR=1.7**								
2015	1397	1015	772	675	625	0.377	0.809	0.926
2020	1430	1003	774	711	656	0.425	0.847	0.922
2025	1453	997	761	735	692	0.458	0.910	0.942
2030	1461	976	742	756	719	0.497	0.969	0.951
2035	1456	936	718	774	738	0.555	1.027	0.953
2040	1443	900	694	792	749	0.604	1.079	0.945
2045	1424	875	665	803	760	0.628	1.143	0.945
2050	1401	838	634	807	767	0.673	1.210	0.951
2055	1373	776	605	804	768	0.769	1.269	0.955
2060	1340	742	580	803	760	0.807	1.309	0.946
2065	1305	727	562	808	743	0.795	1.322	0.920
2070	1269	712	545	808	724	0.782	1.329	0.895
